# Modulation of cortical resting state functional connectivity during a visuospatial attention task in Parkinson's disease

**DOI:** 10.3389/fneur.2022.927481

**Published:** 2022-08-09

**Authors:** Dániel Veréb, Márton Attila Kovács, Szabolcs Antal, Krisztián Kocsis, Nikoletta Szabó, Bálint Kincses, Bence Bozsik, Péter Faragó, Eszter Tóth, András Király, Péter Klivényi, Dénes Zádori, Zsigmond Tamás Kincses

**Affiliations:** ^1^Department of Radiology, Albert Szent-Györgyi Clinical Center, University of Szeged, Szeged, Hungary; ^2^Department of Neurology, Albert Szent-Györgyi Clinical Center, University of Szeged, Szeged, Hungary; ^3^Institute of Diagnostic and Interventional Radiology and Neuroradiology, University Hospital Essen, Essen, Germany

**Keywords:** Parkinson's disease, visual cortex, attention, functional connectivity, fMRI

## Abstract

Visual dysfunction is a recognized early symptom of Parkinson's disease (PD) that partly scales motor symptoms, yet its background is heterogeneous. With additional deficits in visuospatial attention, the two systems are hard to disentangle and it is not known whether impaired functional connectivity in the visual cortex is translative in nature or disrupted attentional modulation also contributes. In this study, we investigate functional connectivity modulation during a visuospatial attention task in patients with PD. In total, 15 PD and 16 age-matched healthy controls performed a visuospatial attention task while undergoing fMRI, in addition to a resting-state fMRI scan. Tensorial independent component analysis was used to investigate task-related network activity patterns. Independently, an atlas-based connectivity modulation analysis was performed using the task potency method. Spearman's rank correlation was calculated between task-related network expression, connectivity modulation, and clinical characteristics. Task-related networks including mostly visual, parietal, and prefrontal cortices were expressed to a significantly lesser degree in patients with PD (*p* < 0.027). Resting-state functional connectivity did not differ between the healthy and diseased cohorts. Connectivity between the precuneus and ventromedial prefrontal cortex was modulated to a higher degree in patients with PD (*p* < 0.004), while connections between the posterior parietal cortex and primary visual cortex, and also the superior frontal gyrus and opercular cortex were modulated to a lesser degree (*p* < 0.001 and *p* < 0.011). Task-related network expression and superior frontal gyrus–opercular cortex connectivity modulation were significantly associated with UPDRSIII motor scores and the Hoehn–Yahr stages (*R* = −0.72, *p* < 0.006 and *R* = −0.90, *p* < 0.001; *R* = −0.68, *p* < 0.01 and *R* = −0.71, *p* < 0.007). Task-related networks function differently in patients with PD in association with motor symptoms, whereas impaired modulation of visual and default-mode network connectivity was not correlated with motor function.

## Introduction

Apart from well-known motor symptoms, the clinical presentation of Parkinson's disease (PD) can include various non-motor deficits as well, involving a range of systems and causing cognitive decline, sleep disorders, or psychoaffective symptoms, among others (1). An increasing number of studies report that the disease also affects the visual system, causing visual deficits such as reduced visual acuity, color vision, or contrast sensitivity (2–5). Although visual deficits are partly because of a loss of dopaminergic cells in the retina, translated changes also appear in the visual cortex. The trajectory of some of these changes not only corresponds to increasing motor deficits, in line with dopaminergic neuron loss (2), but also non-axial symptoms (5) that highlights their heterogeneous nature. Interestingly, a recent study found that therapy-resistant patients with PD who underwent thalamotomy exhibit changes of low-frequency fluctuations in the primary visual cortex that correspond to improving hand tremor symptoms (6). In addition, patients with PD who report visual hallucinations were shown to have abnormal activation patterns in the occipital and temporal extrastriate cortices (7), where structural changes also manifest (8). The involvement of the visual system is accompanied by deficits in the focusing phase of visuospatial attention and an impaired ability to track single or multiple objects, with these symptoms considered to be among the earliest occurring cognitive deficits (5, 9). Visuospatial deficits are fairly selective to the disease related to other neurodegenerative conditions and have been attributed to the dysfunction of the basal ganglia and thalamocortical circuits that also include prefrontal and posterior parietal regions (10), areas that exhibit correlating structural alterations (11). This has since been partly confirmed in the resting state and task-based functional MRI studies. A study found reduced resting state functional connectivity in parietal areas and a consequential over activation in the same areas during the Attention Network Test, while also demonstrating impaired connectivity between areas of task-positive and negative networks (12). A resting-state functional MRI study found that the cerebellum is implicated in visuospatial deficits as well (13). Yet, little is known about how impaired attention and visual function interact in patients with PD. The investigation of this is further hindered by the heterogeneity of resting state functional connectivity changes in PD, since the disease subtype, disease stage, and medication can all have different effects (14–16). It is also unclear whether altered interactions between the attention and visual systems are due to an already impaired baseline connectivity between the two systems, or if there is a component where a related task fails to elicit an increased coordination required for the performance of the task. Studies have so far failed to disentangle primary functional disturbances (translative or otherwise) of the visual system and deficits of attentional modulation arising from more complex alterations in cortical circuitry. In this study, our aim is to examine how a complex visuospatial attention task modulates connectivity irrespective of baseline, possibly heterogeneous resting state functional connectivity changes in a cognitively preserved cohort of patients with PD related to healthy controls. For this, we use task potency, a method that normalizes task-related functional connectivity to a baseline connectivity matrix, usually derived from a resting state scan (17). Although the method originally aims to compare connectivity modulation between different tasks, here it suits our purpose since it enables us to disambiguate purely task-based connectivity changes from heterogeneous baseline resting-state functional connectivity changes observed in PD. In an exploratory manner, we carry out a multivariate analysis to investigate the expression of task-related networks (concentrating on visuospatial attention networks), then proceed with the task potency approach to assess how the task modulates baseline resting-state functional connectivity measures acquired during a different scanning session in the healthy and the patient with PD group.

## Methods

### Task design

**Figure 1:**
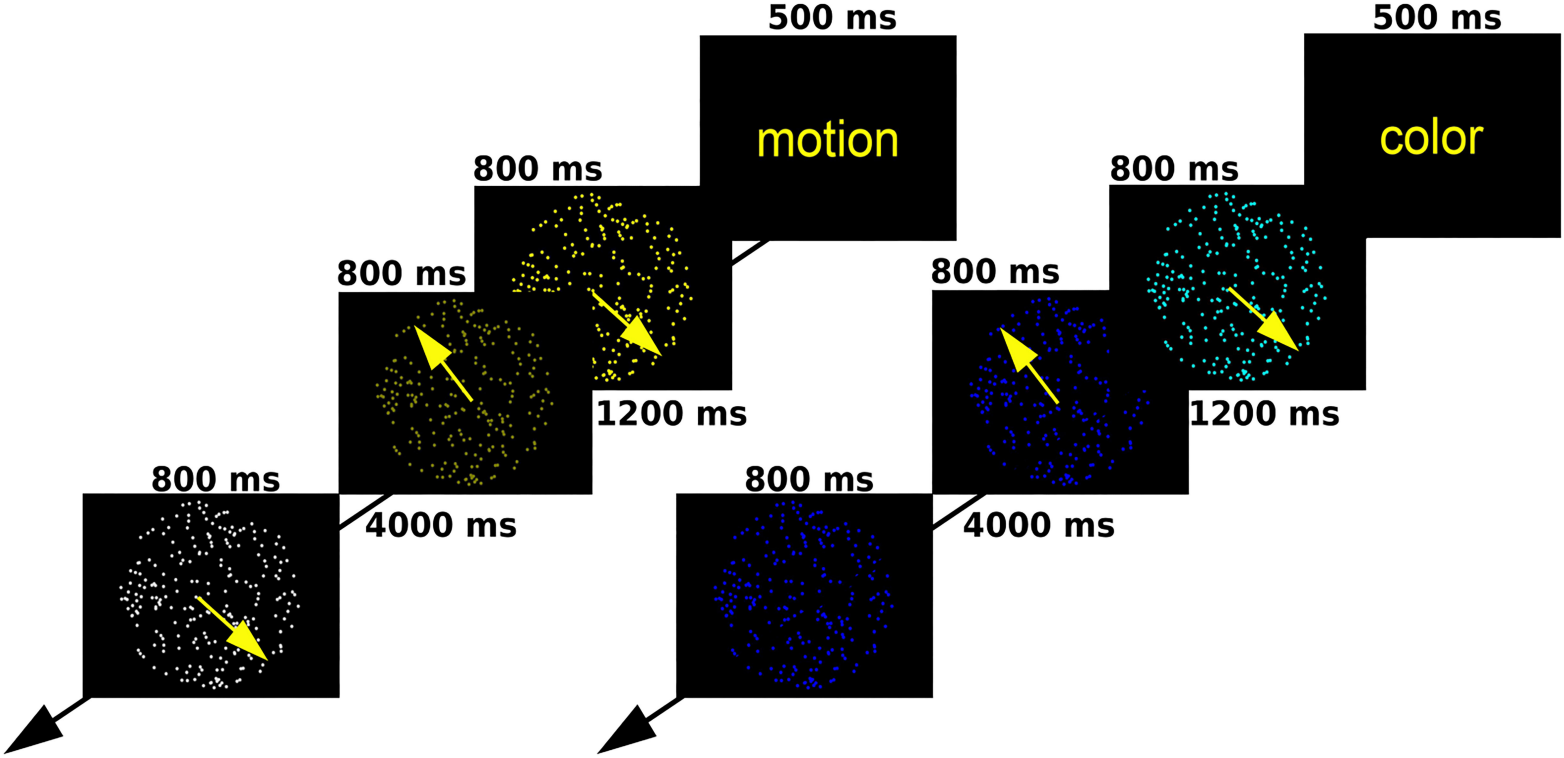
A schematic outline of the moving dots task.

We employed a modified random dot kinematogram paradigm, in which participants attend a circular aperture containing coherently moving dots of a single color (18). At the start of each trial, a text message appeared for 0.5 s, warning participants to attend a specific attribute of consequent stimuli (shade of color or direction of motion). Later, two consecutive random dot kinematograms were displayed for 0.8 s each, followed by a target stimulus after a rest period of 4 s. Participants had to decide whether the relevant attribute of the target was the same as it had been in one of the previous two kinematograms. Each trial lasted for 16.6 s, and there were 20 trials for each condition (40 altogether). Trials were presented in a pseudorandom order so that one condition could occur three times consecutively at maximum. For a depiction of the task, see Figure 1. Participants took part in an offline practice session to ensure they fully understand the task before entering the scanner.

### Participants

In total, 20 patients with a dominantly akinetic-rigid manifestation of idiopathic PD and 20 healthy controls (HCs) were recruited. Patients with PD were only included if they had no dyskinesia. In total, 17 patients with PD and 18 HCs completed the study protocol, and 2 PDs and 2 HCs were later excluded because of the extensive motion during the scans. In total, 16 HCs and 15 PDs were included in the final analysis. Age and biological sex distribution did not differ significantly in the final analyzed population (age—Student's independent samples *t*-test: *p* > 0.05; sex—Fisher's exact test: *p* < 0.12). All the participants included were right-handed. All the participants' eyesight was normal or corrected to normal and none of them reported difficulty performing the task during the practice or scanning session. Healthy controls and patients with PD were cognitively normal (MMSE-score ≥ 27 in all the cases). All the patients with PD except one were on dopaminergic medication [see Table 1 for levodopa equivalent daily dosage (LEDD) scores] and they took their medication before each scan to make sure they are in the ON stage during the scans. All the participants provided their written informed consent as required by the Declaration of Helsinki and the study was approved by the local ethics committee (ref. number 35/2017). Demographic data and clinical characteristics are described in Table 1.

**Table 1:** Demographic data of the participants.

	**Healthy controls**	**Parkinson's disease**
*N*	16	15
Age (mean +/- SD)	64.63 +/- 8.84	65.34 +/- 6.81
Sex (male/female)	8/9	11/4
UPDRSIII-motor (mean +/- SD)	-	25.00 +/- 9.82
Hoehn-Yahr stage (median, range)	-	3 (1–4)
Disease duration (years, mean +/- SD)	-	8.13 +/- 5.82 (1–20)
Levodopa equivalent daily dose (LEDD; mean +/- SD, range)	-	798.27 +/- 483.95 (0–1,800)
Dominant side of motor symptoms (R: right, L: left, B: both)	-	7R,7L,1B

### Image acquisition

Three-dimensional T1-weighted fast-spoiled gradient echo images (FSPGR–IR, TR: 5.3 ms TE: 2.1 TI: 450 ms, slice thickness: 1 mm, matrix: 512 × 512, FOV: 256 × 256 mm^2^, slice no. 312, whole brain coverage, flip angle: 12°) and T2^*^-weighted BOLD EPI images (TR: 2,500 ms, TE: 27 ms, 44 axial slices with 3 mm slice thickness providing whole-brain coverage, FOV: 288 × 288 mm^2^; matrix: 96 × 96, flip-angle: 81°) were acquired on a 3T GE MR750W Discovery scanner (GE, Milwaukee, USA). In total, 270 volumes were acquired during the task fMRI protocol, which took ~12 min. Stimuli were displayed on a screen in the scanner room *via* a video projector. Participants saw the screen through a mirror applied to the head coil frame. Participants also underwent a resting state scan on a separate occasion with the same parameters as the task fMRI, except 240 volumes (10 min) were acquired. Participants were asked to lie motionless with their eyes open and remain awake during the scan. Resting-state and task fMRI scans were conducted on two separate occasions for all the participants, with a difference of 1–3 weeks, at the same time of day.

### Pre-processing

Pre-processing steps were performed *via* FEAT 6.0 as contained in the FMRIB Software Library [FSL (19)], and were the same for resting state and task fMRI scans to ensure comparability. The first 5 volumes were discarded to avoid saturation effects. Motion correction was applied using a rigid body (6 DOF) registration to the middle volume with MCFLIRT. Non-brain tissue was removed from the images *via* FSL's Brain Extraction Tool (20). After a spatial smoothing step with a 6 mm FWHM Gaussian kernel, ICA–AROMA was used to identify and remove motion artifacts (21). In addition, the denoised data underwent nuisance regression to remove the signal from the white matter and cerebrospinal fluid, and high-pass temporal filtering with a 0.01 Hz cut-off. The resulting volumes were normalized to the standard 2 mm MNI-space using a two-stage boundary-based registration process as implemented in FSL.

### Multivariate analysis of task-related activation

We performed a multivariate analysis of the activation maps using tensorial independent component analysis [TICA (22, 23)]. TICA performs a trilinear decomposition of the data into independent component matrices, which describe spatial, temporal, and subject-dependent dimensions. This trilinear combination is optimized *via* a least-squares approach so that different modes are maximally non-Gaussian. MELODIC thresholds spatial maps *via* an alternative hypothesis test based on fitting a Gaussian–gamma mixture model to the distribution of voxel intensities within spatial maps and a posterior probability threshold of *p* > 0.5. The number of independent components was determined automatically using the Laplace approximation to the posterior evidence of the model order. ICs were classified manually as signal or noise based on their spatiotemporal characteristics, adherence to the task design, and uniform expression across the subject pool (no outliers in subject modes). The advantage of TICA to the conventional statistical parametric mapping approach is the increased sensitivity to task-related and background activity during the task that helps discern more subtle alterations, in addition to being more robust to noise arising from head motion (22), a feature convenient in the current study population. Subject modes (referred to as subject scores) from the decomposition were then compared between the patient and healthy cohort to assess the expression of task-related ICs in the two groups using an ordinary least squares approach, correcting for age, and biological sex.

### Analysis of connectivity modulation

Independently from the multivariate analysis, a parcellation-based connectivity analysis was also performed to investigate how resting-state connectivity is modulated during the task. The Schaefer atlas was used to divide the cortex into 100 parcels (24). The inverse of the registration warp fields was used to project atlas ROIs from MNI space to the native space of individual participants for the pre-processed resting state and task fMRI scans. Time courses for each ROI were extracted as the mean of underlying voxel time courses. Then, the partial correlation matrices were calculated independently for the task and resting state data. A Gaussian–gamma mixture model was fit to the connectivity distribution of the resting state partial correlation matrices (25). Employing the approach termed task potency, both resting state and task partial correlation matrices were re-normalized using the parameters of the resting state main Gaussian (connections deemed inactive) to allow valid comparison (17). Modulation of connectivity was calculated by subtracting the resting state (baseline) connection strength from task connection strength. The resulting matrices of connectivity modulation values were thresholded at *t* = ±3.1 to only include connections consistently influenced by the task in the HC group. Baseline resting state connectivity and the extent of modulation in the included connections were compared between groups using a GLM-based approach with a non-parametric permutation test for statistical inference (26). Age and biological sex were included in the model as nuisance regressors. Correction for multiple comparisons was performed by controlling the family-wise error rate. Relationship to clinical variables (UPDRSIII-motor and Hoehn-Yahr stage) was calculated as the partial Spearman's correlation coefficient, corrected for age, and biological sex.

### Data availability

Data supporting the results of this analysis are available on reasonable request from the corresponding author after consideration by the local ethics committee.

## Results

### Tensorial independent component analysis

**Figure 2:**
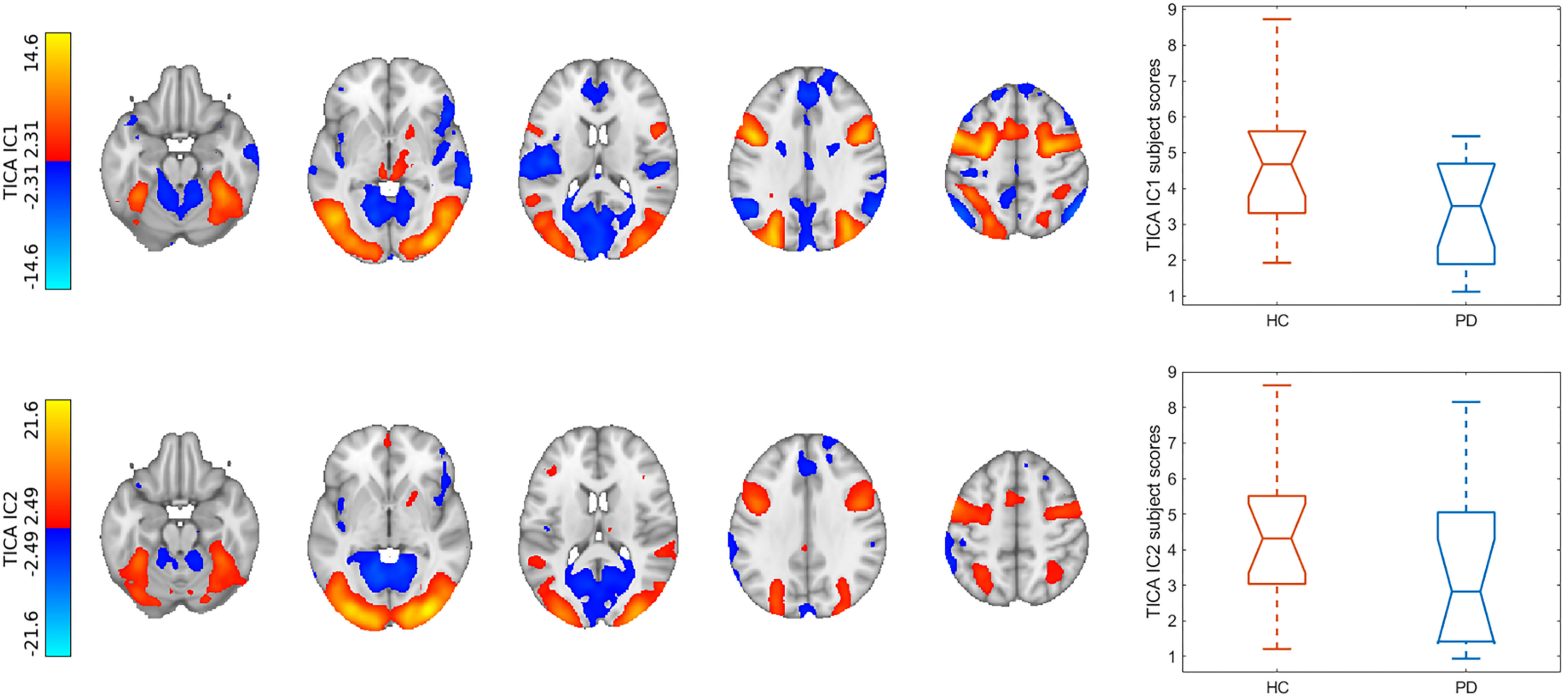
Results of the multivariate analysis. Spatial maps of task-relevant components were upsampled to 1 mm resolution and overlaid on the MNI152 1 mm template. Color bars denote *Z*-statistics. Box plots show group differences in subject modes of corresponding components. TICA, tensorial independent component analysis; IC, independent component; HC, healthy control; PD, Parkinson's disease.

The TICA analysis extracted 50 independent components (ICs), out of which the first 2 were task-relevant networks. The first and second ICs explained 19.6 and 19.41% of the total variance, respectively. IC1 contained the bilateral frontal eye fields, inferior frontal gyri, intraparietal sulci, higher order visual cortices (V3-5), bilateral lingual gyri, thalami, striatum, and superior colliculi which were correlated with the attention part of the task in both conditions [COPE (color): *z* = 20.28, *p* < 0.001; COPE (motion): *z* = 22.00, *p* < 0.001]. Areas of IC1 which anticorrelated with the attention part of the task (and correlated with the recall part) included the primary visual cortices, parietal operculi, dorsal anterior cingulate cortex, anterior insulae, inferior parietal lobules, superior temporal gyri, and the cerebellum. IC2 contained, to a lesser extent, the intraparietal sulci and frontal eye fields, and more prominently, the primary and higher-level visual cortices, and bilateral thalami. IC1 subject scores were significantly higher in the healthy group (*p* < 0.027), adjusted for age and biological sex (see Figure 2).

### Connectivity modulation

**Figure 3:**
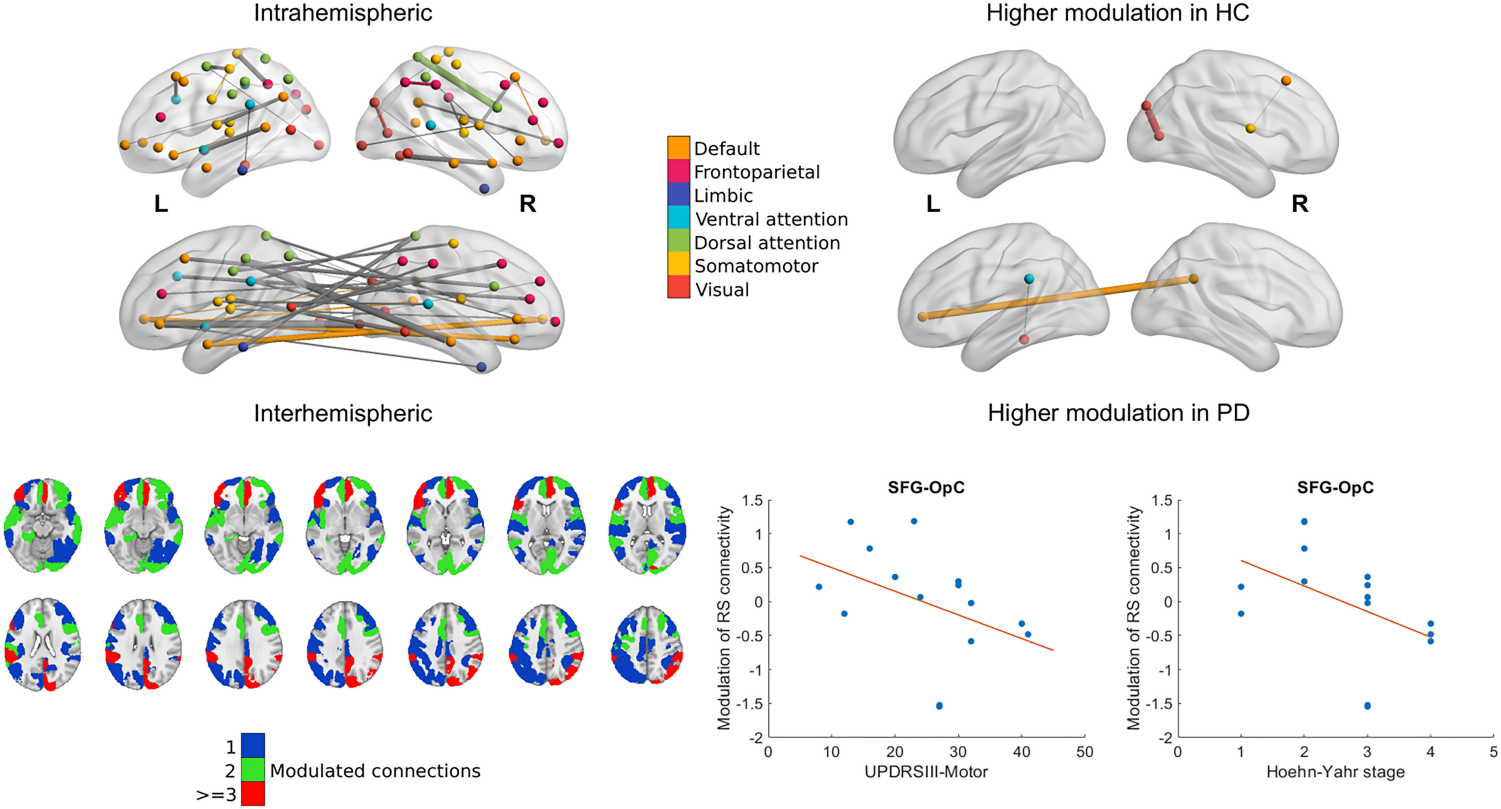
Modulation of cortical resting-state functional connectivity during the task. The 3D representations show relevant intra- and interhemispheric connections that were consistently modulated during the task in the healthy group (marked by a change with an effect size of at least *t* = 3.1 in connectivity; left) or were modulated differently in the healthy and PD cohort (right). Nodes and within-network edges are color-coded according to their overlap with the 7-network parcellation in (27); between-network edges are shown in gray. Axial slices show parcels from the Schaefer atlas with at least 1 connection modulated consistently group-wise during the task. Parcel ROIs were overlaid on the MNI152 1 mm brain template and are shown in neurological orientation. Scatter plots show the statistically significant association between clinical characteristics and modulation of the superior frontal gyrus (SFG)–opercular cortex (OpC) connection in the PD cohort with a least-squares line superimposed.

Baseline resting state connectivity showed no differences between the two groups after correcting for multiple comparisons. Mainly, nodes belonging to the visual frontoparietal, dorsal attention, ventral attention, default mode, and somatomotor networks were consistently modulated during the task in the healthy cohort exemplified by a one-sample *t*-statistic of at least *t* = ±3.1 (*p* < 0.001). The connection between the right primary visual cortex and parietooccipital cortex, and also the connection between the right superior frontal gyrus and right opercular cortex was modulated to a significantly smaller degree in the PD cohort (*p* < 0.001 and *p* < 0.013, corrected for multiple comparisons). The connection between the right precuneus and the left ventromedial prefrontal cortex, and also the connection between the left parietal operculum and lingual gyrus was modulated to a significantly higher degree in the PD cohort (*p* < 0.004 and *p* < 0.011, corrected for multiple comparisons; see Figure 3).

### Relationship to clinical characteristics

Subject scores of both IC1 and IC2 correlated significantly with UPDRSIII-motor scores and the Hoehn–Yahr stages (IC1: *R* = −0.72, *p* < 0.006 and *R* = −0.90, *p* < 0.001; IC2: *R* = −0.72, *p* < 0.006 and *R* = −0.89, *p* < 0.001). Lower modulation of right superior frontal gyrus-right opercular cortex connectivity was associated with higher UPDRSIII-motor scores and the Hoehn–Yahr stages (*R* = −0.68, *p* < 0.01; *R*= −0.71, *p* < 0.007; see Figure 3). We observed no correlations with the LEDD scores.

## Discussion

In this study, we report altered activity of task-related networks during a visuospatial attention task in non-demented patients with PD that correlate with clinical status. Apart from disrupted activation patterns, we found that parietooccipital, prefrontal, and default-mode network connections are modulated differently during the task. Parietooccipital and default-mode network connectivity modulation did not correlate with clinical status, whereas modulation of superior frontal gyrus—opercular cortex connectivity decreased with the higher UPDRSIII-scores and the Hoehn–Yahr stage. Visuospatial deficits have been recognized as fairly specific and early alterations in the Parkinson's disease, and there are several reports of altered brain activity during visuospatial tasks, even though results are somewhat heterogeneous. A recent study investigated activation during the Attention Network Test and found increased activation in the right frontal eye field, bilateral intraparietal sulci, and precuneus in non-demented patients with PD, suggesting that impaired attention might be due to diminished coordination between the default mode and task-positive networks (12). Another study reported decreased activation in the middle frontal gyrus and inferior parietal lobule (IPL) during a visuospatial N-back task in patients with PD with and without cognitive impairment (28). However, there are reports that baseline perfusion changes might occur in parietal regions even in non-demented patients with PD, which can potentially affect activation-based studies (29). We attempted to circumvent this by employing a network-oriented approach, tensorial independent component analysis, which has the additional advantage of being less sensitive to head motion, another possible confounding factor that is especially important in the investigated cohort (30). Our results partly confirm previous accounts, since, we found that the healthy pattern of task-positive networks (which, in this case, mainly involve the visual and dorsal attention networks) is altered in the PD cohort. Although there are significantly fewer papers on task-related functional network connectivity in PD, our results support the notion of a functional task-related network reorganization during complex cognitive tasks (31). A further complication in the investigation of task-related networks is that there are prominent and widespread resting state functional connectivity alterations in Parkinson's disease (32), although these might not be sufficient to fully characterize connectivity alterations apart from a tentative consensus on default-mode network alterations (33). Indeed, in this study, we found no differences in full and partial correlation-based functional connectivity in patients with PD during the resting state. Since the architecture of task state networks is highly dependent on and similar to resting-state connectivity (34), it is unclear in this case whether the observed task-related changes are because of an already impaired resting state baseline, or to different modulation of baseline connectivity. To mitigate this, we employed the task potency method, an approach that uses mixture modeling to subtract baseline connection strength from connection strength observed during the task, thus, providing a general measure of connectivity modulation (17). This approach suited our objectives better than generalized psychophysiological interactions (35) or dynamic causal model-based analyses (36) since we included multiple conditions to account for different visual features known to be affected in PD (color and motion) and aimed to summarize modulation differences in an exploratory manner over the whole cortex. With this approach, we found several diverging results. Diminished modulation of superior frontal gyrus–opercular cortex was associated with a more advanced clinical condition. The superior frontal region is involved in motor coordination, planning and control, and also attention *via* the frontal eye field area (37) and working memory (38), and the frontal operculum is also involved in the ventral attention system (39). Both regions are also involved in dopaminergic function *via* the mesocortical pathway and striato–thalamo–cortical loops (40), which might explain the association with the motor symptoms. In addition, we found that the connection between the precuneus and the ventromedial prefrontal cortex was modulated by the task to a significantly higher degree in the cohort with PD. This is in line with the previous accounts of impaired deactivation of the default mode network during visuospatial tasks in PD and might point to a failure of the default mode network to disengage when faced with goal-oriented task demands (12). Furthermore, connectivity between the right primary visual cortex and parietooccipital cortex was modulated to a significantly smaller degree in the cohort with PD, with no association with motor symptoms. One explanation for this could be that, potentially, the ability of spatial attention to enhance visual cortex excitability, thus, facilitating the processing of favored stimuli is impaired (41). This would mean that, apart from translative changes arising from the dopaminergic dysfunction in the anterior visual system, attentional modulation of the visual process is also impaired, either by downstream effects of complex cortical network changes or *via* more specific disease-related pathological alterations. Our study has some limitations. Furthermore, studies investigating a larger cohort are required to test for more subtle alterations in connectivity modulation of the visuospatial attention system. Also, here, we did not explicitly test patients with PD for color vision or motion perception, however, they reported no difficulty in performing the task and performed similarly to the healthy group, which indicates they had no color vision or motion perception impairments.

## Conclusion

In this study, we demonstrate that task-related networks function differently in non-demented patients with PD during a visuospatial attention task, and the task modulates functional connectivity differently in patients with PD irrespective of baseline resting state connectivity alterations. Modulation of prefrontal connectivity was strongly associated with motor symptoms and clinical parameters, while modulation of default mode network and parietooccipital connectivity was not related to the patients' clinical condition. These results suggest that visuospatial dysfunction does not exclusively arise from the impairment of the visual system, but incorporates more complex effects of attentional modulation and cortical network changes.

## Data availability statement

The datasets presented in this article are not readily available because they contain sensitive clinical and personal details; anonymized data will be shared by the corresponding author upon reasonable request, after consideration by the local ethics committee. Requests to access the datasets should be directed to ZK, kincses.zsigmond.tamas@szte.hu.

## Ethics statement

The studies involving human participants were reviewed and approved by Regional Human Biomedical Research Ethics Committee, University of Szeged, Szeged, Hungary. The patients/participants provided their written informed consent to participate in this study. Written informed consent was obtained from the individual(s) for the publication of any potentially identifiable images or data included in this article.

## Author contributions

DV: study conceptualization, data acquisition, data analysis, and writing the manuscript. MK and SA: data analysis and writing the manuscript. KK: data acquisition and data analysis. NS: study conceptualization and data acquisition. BK, BB, PF, ET, and AK: data acquisition. PK and DZ: data acquisition and reviewing the manuscript. ZK: study conceptualization, study supervision, and reviewing the manuscript. All authors contributed to the article and approved the submitted version.

## Funding

The study was supported by a Horizon 2020 Grant (H2020-MSCA-RISE-2016 734718). PF was supported by the National Research, Development and Innovation Office–NKFIH Grant (No. FK 135870). DV, NS, and BK were supported by the National Research, Development and Innovation Office–NKFIH grant (No. K 139415).

## Conflict of interest

The authors declare that the research was conducted in the absence of any commercial or financial relationships that could be construed as a potential conflict of interest.

## Publisher's note

All claims expressed in this article are solely those of the authors and do not necessarily represent those of their affiliated organizations, or those of the publisher, the editors and the reviewers. Any product that may be evaluated in this article, or claim that may be made by its manufacturer, is not guaranteed or endorsed by the publisher.
